# Supplementary material for: Complexome profiling of the Chlamydomonas psb28 mutant reveals TEF5 as an early PSII assembly factor
Source: Plant Cell. 2025 Mar 17;37(6):koaf055. doi: 10.1093/plcell/koaf055 (PMC12164590; doi:10.1093/plcell/koaf055)
Supplement: koaf055_Supplementary_Data [file koaf055_supplementary_data.zip › psb28_interactive.zip!/plots/intensity/qualityPlots/peptides/TATA_Cre10.g438550.t1.2.html]

### Cre10.g438550.t1.2

Trivial name: TATA  
SeqCoverage [median]: 31.60  
Mass: 11.87 kDa  
  
 

| Sequence | MissedCleavage | Mass [Da] | wtIntensity | muIntensity | Score |
| --- | --- | --- | --- | --- | --- |
| AGMAADDAKKPAAVEEK | 2 | 1700.84 | 1788.95 | 1320.98 | 84.94 |
| KPAAVEEKKEETK | 3 | 1485.80 | 4242.58 | 6711.43 | 131.32 |
| SFQTAASEFNDELK | 0 | 1585.73 | 4491.14 | 18832.39 | 201.00 |
| SFQTAASEFNDELKAGMAADDAK | 1 | 2416.09 | 2963.15 | 10349.77 | 103.67 |
|  |  |  | 13485.8 | 37214.6 |  |
